# Identification of a Rare and Potential Pathogenic *MC4R* Variant in a Brazilian Patient With Adulthood-Onset Severe Obesity

**DOI:** 10.3389/fgene.2020.608840

**Published:** 2020-12-09

**Authors:** Kaio Cezar Rodrigues Salum, Guilherme Orofino de Souza, Gabriella de Medeiros Abreu, Mário Campos Junior, Fabiana Barzotto Kohlrausch, João Regis Ivar Carneiro, José Firmino Nogueira Neto, Fernanda Cristina C. Mattos Magno, Eliane Lopes Rosado, Lohanna Palhinha, Clarissa Menezes Maya-Monteiro, Giselda Maria Kalil de Cabello, Pedro Hernán Cabello, Patrícia Torres Bozza, Verônica Marques Zembrzuski, Ana Carolina Proença da Fonseca

**Affiliations:** ^1^Human Genetic Laboratory, Department of General Biology, Institute of Biology, Federal Fluminense University, Niterói, Brazil; ^2^Human Genetics Laboratory, Oswaldo Cruz Institute, Oswaldo Cruz Foundation, Rio de Janeiro, Brazil; ^3^Clementino Fraga Filho University Hospital, Federal University of Rio de Janeiro, Rio de Janeiro, Brazil; ^4^Department of Pathology and Laboratory, Rio de Janeiro State University, Rio de Janeiro, Brazil; ^5^Institute of Nutrition Josué de Castro, Federal University of Rio de Janeiro, Rio de Janeiro, Brazil; ^6^Laboratory of Immunopharmacology, Oswaldo Cruz Institute, Oswaldo Cruz Foundation, Rio de Janeiro, Brazil; ^7^Human Genetics Laboratory, Grande Rio University, Rio de Janeiro, Brazil

**Keywords:** severe obesity, *MC4R*, non-syndromic monogenic obesity, adulthood-onset obesity, mutation

## Abstract

**Background:**

The melanocortinergic pathway orchestrates the energy homeostasis and impairments in this system often lead to an increase in body weight. Rare variants in the *melanocortin 4 receptor* (*MC4R*) gene resulting in partial or complete loss of function have been described with autosomal co-dominant inheritance. These mutations are the most common cause of non-syndromic monogenic obesity. In this context, this study aimed to sequence the *MC4R* gene in a Brazilian cohort of adults with severe obesity.

**Methods:**

This study included 163 unrelated probands with Body Mass Index (BMI) ≥ 35 kg/m^2^, stratified into three groups, according to the period of obesity onset. From the total sample, 25 patients were enrolled in the childhood-onset group (0–11 years), 19 patients in the adolescence/youth-onset group (12–21 years), and 119 patients in the adult-onset group (>21 years). Blood pressure, anthropometric and biochemical characteristics were obtained, and the *MC4R* coding region of each subject’s DNA was assessed using automated Sanger sequencing.

**Results:**

Significant anthropometric differences between the groups were observed. Higher body weight and BMI medians were found in patients with childhood-onset or adolescence/youth-onset when compared to the adulthood-onset obesity group. A total of five mutations were identified, including four missense variants: p.Ser36Thr, p.Val103Ile, p.Ala175Thr, and p.Ile251Leu. Additionally, we observed one synonymous variant (p.Ile198=). The p.Ala175Thr variant was identified in a female case with severe obesity and adulthood-onset. This variant was previously described as a partial loss-of-function mutation, in which the minor allele poses dominant-negative effect, probably resulting in reduced cAMP activity.

**Conclusion:**

This study showed a prevalence of common and rare variants in a cohort of Brazilian adults with severe obesity and candidates to bariatric surgery. We have identified a rare potentially pathogenic *MC4R* variant in a Brazilian patient with severe and adulthood-onset obesity.

## Introduction

Worldwide Health Organization (WHO) defines obesity as excessive body fat mass accumulation, which may impair health. This disease is defined by a body mass index (BMI) > 30 kg/m^2^ in adults (>18 years old) (World Health Organization, 2018). The prevalence of obesity has been increasing since the past four decades, impacting developed and developing countries (Canhada et al., 2019). In 2016, 650 million adults and 1,234 million children and adolescents were described as obese in the world (World Health Organization, 2018). In some middle-income countries, obesity represents the leading public health problem (Ng et al., 2014). Brazil belongs to a group of 10 countries that together concentrate more than 50% of obese population in the world (Ng et al., 2014). It was estimated that 19.8% of Brazilian subjects were obese in 2016, and this frequency was related to age and education level (Brasil, 2019). In 2011, the Brazilian public health system spent 269.6 million dollars to treat patients with obesity and/or obesity-related disorders (de Oliveira et al., 2015).

The excess of fat mass accumulation is caused by an impaired equilibrium between energy intake and expenditure (Canhada et al., 2019). However, there is a notable difference in individual phenotypic expression between subjects sharing the same environment. This variability occurs due to the multifactorial etiology of obesity, resulting from a complex interaction between cultural, nutritional, and environmental factors with interindividual genetic variability (Albuquerque et al., 2017; Meldrum et al., 2017; Velázquez-Fernández et al., 2017; Qasim et al., 2018; Narisada and Suzuki, 2019). Studies using families, twins, and adopted subjects have investigated the genetic contribution in obesity, and a heredity of 70% was estimated for BMI (Silventoinen et al., 2010; Albuquerque et al., 2015; Thaker, 2017). Polygenic obesity is caused by the interplay among many common genetic variants, and is the most common form of this disease (Farooqi and O’Rahilly, 2006). However, monogenic obesity, caused by mutations in a single gene, is a rare but severe form of the disease, associated with 5% of the cases (da Fonseca et al., 2017; Fonseca et al., 2019).

The hypothalamus plays a pivotal role in the energy homeostasis through the leptin-melanocortin pathway. Several variants at protein-coding genes related to this pathway have been described in obese subjects (da Fonseca et al., 2017). *Melanocortin 4 receptor* (*MC4R*) gene mutations are related to approximately 6% of obesity cases, being the most common causes of non-syndromic monogenic obesity (Farooqi et al., 2003).

*MC4R* gene is located on chromosome 18q21.3, spans more than 1.6 kb, and encodes a 332 amino acid protein. This protein is expressed in several areas of the brain, including the paraventricular nucleus of the hypothalamus, which has an essential role in energy homeostasis. Activation of MC4R by α-melanocyte-stimulating hormone (α-MSH) increases the satiety signal and suppresses food intake, regulating energy balance and body weight (Morton et al., 2006; Larder et al., 2014). Animal-based studies showed that *Mc4r*-deficient mice developed obesity associated with an increase in linear growth, hyperphagia, hyperinsulinemia, and hyperglycemia (Huszar et al., 1997; Marie et al., 2000).

*MC4R* genetic variants are inherited in an autosomal co-dominant manner (Farooqi et al., 2003; Farooqi, 2015). Several deleterious mutations were identified in this gene, which could result in partial or complete loss of protein function (Collet et al., 2017b). The prevalence of these pathogenic variants varies among ethnicities, ranging from 0.5 to 5% in children with severe obesity (Miraglia Del Giudice et al., 2002; Melchior et al., 2012; Saeed et al., 2015). The first *MC4R* variant related to monogenic obesity was a frameshift mutation (c.631_634delCTCT), identified in two heterozygous patients, a 4-years-old boy and his father with early-onset obesity. The proband’s mother did not carry the variant and was not obese (Yeo et al., 1998). Another frameshift mutation (c.732_735dupGATT) was identified in a family with early-onset obesity history. The mutation was co-segregated with the severe obesity phenotype over three generations (Vaisse et al., 1998). Consistently, loss-of-function mutations in *MC4R* gene cause increased appetite and severe early-onset obesity and some patients can also exhibit hyperinsulinemia and increased linear growth (Farooqi et al., 2003; Melchior et al., 2012; Doulla et al., 2014).

Recently, our group identified an *MC4R* start lost mutation (p.Met1?) in a female adult patient with childhood-onset obesity, moderate binge-eating disorder, and high caloric intake (Fonseca et al., 2019). Here, we extend the screening of the *MC4R* gene in new Brazilian patients with severe obesity to identify other pathogenic variants. Therefore, this study aimed to investigate the prevalence of *MC4R* variants in a Brazilian cohort of severely obese patients with different periods of disease onsets (childhood, adolescence/youth-onset, or adulthood).

## Materials and Methods

### Study Population

This cross-sectional observatory study included 163 unrelated probands of both genders (88.3% female), aged from 18 to 65 years old [median of 41 (33; 49)], from Rio de Janeiro, Brazil. The selection criteria were patients with severe obesity (BMI ≥ 35 kg/m^2^). Pregnant or lactating women, subject in use of weight management-related medications, and individuals with the presence of symptoms suggestive of obesity-related syndromes were excluded. The cohort was stratified into three groups according to the self-reported period of obesity onset. The childhood-onset group (0–11 years) comprised 25 patients, the adolescence/youth-onset group (12–21 years) 19 patients, and the adult-onset group (>21 years) 119 patients. All volunteers were recruited from a non-governmental organization called Rescue Group to Self-Esteem and Citizenship of the Obese (in Portuguese, “Grupo de Resgate à Autoestima e Cidadania do Obeso”). This study protocol was approved by the Oswaldo Cruz Foundation Ethics Committee (CAAE: 09225113.0.0000/Protocol n°: 346.634). Written Informed consent was obtained from all participants (resolution n° 466/2012 of Ministry of Health, Brazil).

### Anthropometric and Biochemical Analysis

Height, body weight, hip, waist, and neck circumference and blood pressure were measured as described previously (da Fonseca et al., 2019). These measurements were used to calculate the body adipose index (BAI), BMI, and waist-hip ratio (WHR) of each patient.

Following an overnight fast, a blood sample of each individual was collected and the levels of glucose, total cholesterol (TC), high-density lipoprotein cholesterol (HDL-c), and triglycerides (TG) were assessed through the oxidase-peroxidase method (BioSystems, Barcelona, Spain). The Friedewald equation was used to calculate the low-density lipoprotein cholesterol (LDL-c) levels. Glycated hemoglobin (HbA1c) levels were measured by turbidimetric inhibition immunoassay (TINIA), and latex agglutination assay was used to evaluate the levels of C-reactive protein (CRP). Patients using medications with effects in any of these parameters were not considered in the statistical analysis for the specific trait (blood pressure = 31; glucose = 16).

#### *MC4R* Gene Screening

Genomic DNA extraction was performed from peripheral blood using the commercial QIAamp Blood kit (Qiagen, Valencia, CA, United States). Two pairs of primers were designed with Primer3Plus software and used for screening the *MC4R* coding region spanning >1.6 kb by the Sanger Automatic Sequencing Method. The primer sequences are described elsewhere (Fonseca et al., 2019). Polimerase Chain Reaction (PCR) was performed in 25 μl final volume, including 10–20 ng of DNA, 1 unit of Taq Brasil (Invitrogen, Carlsbad, CA, United States), 10× PCR Buffer, 0.2 mmol/L of each dNTP, 0.3 pmol of each primer and 5.0 mmol/L MgCl_2_. PCR conditions were the same for both pair of primers: 94°C for 3 min, followed by 35 cycles of 94°C for 45 s, 64°C for 30 s and 72°C for 1 min and 30 s; and a final extension of 72°C for 10 min. The PCR products were verified in 1% agarose gels and purified with ExoSAP kit (Thermo Fisher Scientific, Waltham, MA, United States).

The sequencing reaction was performed with Big Dye Terminator v3.1 in a final volume of 10 μl, including 10–40 ng of purified PCR products, 1× sequencing buffer, 1.0 μl of Big Dye, and 0.32 pmol of primers. Sequencing conditions were 40 cycles of 94°C for 10 s, 50°C for 5 s, and 60°C for 4 min. The sequences were analyzed and aligned with a reference sequence available at the National Center of Biotechnology Information (access number: NM_005912.2), using BioEdit Sequence Alignment Editor software version 7.2.5. The detected mutations were confirmed by bidirectional sequencing in a second PCR reaction, and the potential pathogenicity of missense variants was verified using Polyphen (Polymorphism Phenotyping), SIFT (Sorting Intolerant from Tolerant), PhD_SNP (Predictor of human Deleterious Single Nucleotide Polymorphisms), SNAP (Screening for non-acceptable polymorphisms), and PANTHER (Protein Analysis Through Evolutionary Relationships) softwares. In summary, these tools use available data of sequence, structural and/or functional annotations to predict the effect of amino acid substitutions on protein structure and function. Additionally, we also classified the genetic variants following the American College of Medical Genetics and Genomics (ACMGG) Standards and guidelines for the interpretation of sequence variants (Richards et al., 2015). The effect of single amino acid change in the protein stability was also assessed using I-Mutant 2.0^1^ and Mupro Tool^2^ softwares based on machine learning method. We also investigated whether the variants detected in our study were previously described in public databases, including PubMed, Clinvar, dbSNP^3^, Genome Aggregation Database (gnomAD)^4^, 1000 Genomes project database (1,000 genomes)^5^ and Online Archive of Brazilian Mutations (ABraOM)^6^. Evolutionary conservation of wild type amino acid was tested with multiple sequence alignment by Clustal Omega (1.2.4)^7^.

### Statistical Analysis

Descriptive data were shown as median (IQR 25–75%) and frequency (%). Kruskal-Wallis and chi-squared statistical tests were used to compare quantitative and qualitative variables, respectively. Statistical analyses were performed in the SPSS software package version 20.0 (SPSS, Chicago, IL, United States). The significance level was set at *P* < 0.05.

## Results

### Anthropometric, Blood Pressure, and Biochemical Characteristics

A total of 163 obese patients [BMI median: 44.3 (39.3; 50.1) kg/m^2^; age median: 41 (33; 49) years old] were enrolled and had their anthropometric, biochemical profile, and blood pressure parameters characterized and summarized in Table 1. The childhood-onset group was composed by 96.0% of female patients, with a median age of 37 years old, median weight of 110.2 kg, median BMI of 40.5 kg/m^2^ and median BAI of 47.1%. The adolescence/youth-onset group had 84.2% female cases, median age of 31 years old, median weight of 113.5 kg, median BMI of 42.4 kg/m^2^, and median BAI of 50.4%. Finally, the adult-onset group had 87.4% female subjects, median of age of 44 years old, median weight of 120.2 kg, median BMI of 45 kg/m^2^, and median BAI of 49.1%. We also observed that individuals with childhood-onset obesity presented higher prevalence of hypertension.

**TABLE 1:** Anthropometric and serum biochemistry profile characterization of the study cohort according to onset obesity.

**Variables**	**All**		**Childhood-onset obesity**		**Adolescence/youth-onset obesity**		**Adult-onset obesity**		***p*-value**
	**n**	**Values**	**n**	**Values**	**n**	**Values**	**n**	**Values**	
Age (years)	163	41.0 (33.0; 49.0)	25	37.0 (26.5; 46.5)	19	31.0 (27.0; 35.0)	119	44.0 (36.0; 51.0)	<0.001
Gender									
Female	163	144 (88.3)	25	24 (96.0)	19	16 (84.2)	119	104 (87.4)	0.398
Male		19 (11.7)		1 (4.0)		3 (15.8)		15 (12.6)	
Weight (kg)	163	118.3 (104.5; 134.0)	25	110.2 (101.3; 127.9)	19	113.5 (101.7; 127.0)	119	120.2 (105.0; 136.0)	0.173
Height (m)	163	1.62 (1.57; 1.68)	25	1.63 (1.59; 1.69)	19	1.63 (1.59; 1.66)	119	1.62 (1.57; 1.68)	0.743
BMI (kg/m2)	163	44.3 (39.3; 50.1)	25	40.5 (37.9; 46.9)	19	42.4 (37.4; 49.0)	119	45.0 (40.0; 51.9)	0.060
BAI (%)	161	49.0 (42.1; 54.5)	25	47.1 (41.0; 51.7)	18	50.4 (41.6; 52.1)	118	49.1 (42.8; 55.7)	0.291
Waist circumference (cm)	161	128.0 (117.5; 140.0)	25	122.0 (110.5; 132.5)	18	116.0 (107.4; 128.0)	118	130.0 (120.0; 143.6)	0.001
Hip circumference (cm)	161	137.0 (127.1; 150.0)	25	132.5 (125.0; 143.5)	18	133.5 (123.2; 146.2)	118	140.0 (128.0; 150.6)	0.172
WHR	161	0.92 (0.87; 0.99)	25	0.90 (0.83; 0.97)	18	0.87 (0.81; 0.93)	118	0.94 (0.88; 0.99)	0.005
SBP (mm Hg)	78	123.5 (110.7; 139.0)	6	126.5 (102.0; 132.0)	11	115.0 (104.0; 129.0)	61	124.0 (114.5; 149.5)	0.174
DBP (mm Hg)	78	80.0 (72.7; 90.0)	6	84.5 (59.7; 104.5)	11	74.0 (69.0; 87.0)	61	81.0 (74.5; 90.0)	0.482
FGP (mg/dL)	123	96.0 (90.0; 109.0)	20	95.0 (92.0; 101.0)	19	92.0 (87.0; 109.0)	84	98.0 (90.2; 112.0)	0.315
Cholesterol total (mg/dL)	138	193.0 (164.7; 227.2)	23	185.0 (160.0; 211.0)	19	181.0 (161.0; 211.0)	96	197.5 (167.0; 231.7)	0.173
HDL-cholesterol (mg/dL)	138	47.0 (42.7; 53.0)	23	44.0 (40.0; 51.0)	19	47.0 (43.0; 49.0)	96	48.5 (43.0; 54.0)	0.177
LDL-cholesterol (mg/dL)	136	120.5 (96.2; 145.0)	23	117.0 (98.0; 128.0)	19	119.0 (95.0; 132.0)	94	121.0 (94.5; 152.0)	0.470
Tryglicerides (mg/dL)	138	121.0 (92.0; 160.5)	23	97.0 (78.0; 140.0)	19	117.0 (80.0; 188.0)	96	124.0 (99.2; 167.7)	0.106
HbA1c (%)	84	5.8 (5.2; 6.4)	8	5.7 (5.3; 6.7)	9	5.3 (4.3; 6.2)	67	5.8 (5.3; 6.4)	0.413
CRP (mg/dL)	82	0.97 (0.50; 1.46)	8	0.94 (0.54; 1.90)	9	0.72 (0.20; 1.03)	65	0.98 (0.52; 1.61)	0.308
Metabolic syndrome									
Yes	125	40 (32.0)	14	4 (28.6)	12	7 (58.3)	99	29 (29.3)	0.120
No		85 (68.0)		10 (71.4)		5 (41.7)		85 (70.7)	
Hypertension									
Yes	155	59 (38.1)	25	13 (52.0)	11	9 (81.8)	119	37 (31.1)	0.001
No		96 (61.9)		12 (48.0)		2 (18.2)		82 (68.9)	

*p-value for differences among childhood-, adolescence/youth- and adult-onset obesity.BAI, body adiposity index; BMI, body mass index; CRP, C-reactive protein; DBP, diastolic blood pressure; FPG, Fasting Plasma Glucose; HDL-cholesterol, high-density lipoprotein-cholesterol; LDL-cholesterol, low-density lipoprotein-cholesterol; HbA1c, glycated hemoglobin; SBP, systolic blood pressure; WHR, waist–hip ratio.*

### *MC4R* Screening Results

*MC4R* gene sequencing analysis revealed the presence of five variants. Four of them were missense mutations (p.Ser36Thr, p.Val103Ile, p.Ala175Thr, and p.Ile251Leu), and one was a synonymous variant (p.Ile198=) (Table 2). In our 44 cases with early-onset obesity, we have identified two patients with p.Val103Ile polymorphism and one subject with p.Ile198= alteration. No pathogenic variation was identified in our new patients.

**TABLE 2:** Variants identified in the *MC4R* screening in Brazilian cohort.

**dbSNP ID**	**Protein level**	**cDNA level**	**Mutation type**	**MAF gnomAD**		**MAF 1000 genomes**	**MAF ABraOM**	**MAF our study**	***n***	**Onset of obesity**
				**Exomes**	**Genomes**					
rs954123325	p.Ser36Thr	c.106T > A	Missense	3.97804^–06^	3.18492^–05^	NA	NA	0.003	1	Adult
rs2229616	p.Val103Ile	c.307G > A	Missense	0.016	0.016	0.016	0.010	0.022	6	Childhood
rs121913563	p.Ala175Thr	c.523G > A	Missense	0.0005	0.0002	NA	NA	0.002	1	Adult
rs61741819	p.Ile198=	c.594C > T	Synonymous	0.003	0.012	0.013	0.002	0.003	1	Adult
rs52820871	p.Ile251Leu	c.751A > C	Missense	0.007	0.007	0.003	0.008	0.002	1	Adult

*N, number of patients; MAF, minor allele frequency; NA, data not available; SNP, single nucleotide polymorphism.*

Regarding the patients with adulthood-onset obesity, we identified six subjects with p.Val103Ile (one homozygous), one carrier of p.Ile198=, and one subject carrying the p.Ser36Thr variant. We also identified two alterations that were not previously observed in our Brazilian cohort (p.Ile251Leu and p.Ala175Thr) (Fonseca et al., 2019). The identified variants were assessed using prediction tools, which did not classify our variants as pathogenic. However, the ClinVar database and ACMGG Standards and guidelines classified the p.Ala175Thr as a pathogenic variant (Table 3).

**TABLE 3:** Predicted significance of the variants identified in the *MC4R* screening.

**Variant**	**Variant effect**							**Protein stability**	
	**Polyphen**	**SIFT**	**PhD_SNP**	**SNAP**	**PANTHER**	**ClinVar**	**ACMGG standards and guidelines**	**I-mutant 2.0**	**Mupro Tool**
p.Ser36Thr	Benign	Tolerated	Neutral	Neutral	Probably Benign	NA	Likely benign	Decreased	Decreased
p.Val103Ile	Benign	Tolerated	Neutral	Effect	Probably Benign	Likely benign	Likely benign	Decreased	Decreased
p.Ala175Thr	Benign	Tolerated	Neutral	Neutral	Probably Benign	Pathogenic	Pathogenic	Decreased	Decreased
p.Ile198=	NA	NA	NA	NA	NA	Benign	Likely benign	NA	NA
p.Ile251Leu	Benign	Tolerated	Neutral	Neutral	Probably Benign	Likely benign	Likely benign	Decreased	Decreased

*NA, data not available; Polyphen, Polymorphism Phenotyping; SIFT, Sorting Intolerant from Tolerant; PhD_SNP, Predictor of human Deleterious Single Nucleotide Polymorphisms; SNAP, Screening for non-acceptable polymorphisms; PANTHER, Protein Analysis Through Evolutionary Relationships; ACMGG Standards and Guidelines, American College of Medical Genetics and Genomics Standards and Guidelines.*

### The Phenotype of p.Ser36Thr and p.Ala175Thr Carries

The p.Ser36Thr variant carrier is a 42-year-old female patient with morbid obesity. At the moment of clinical examination, her anthropometric data were: weight, 123.6 kg; height, 1.63 m; BMI, 46.5 kg/m^2^; waist circumference, 142.5 cm; hip circumference, 149 cm; neck circumference, 40 cm; BAI, 53.6% and WHR, 0.96. The blood pressure was 99/62 mm Hg. Biochemical analysis showed that fasting blood glucose (FBG) was 69 mg/dL; TC, 153 mg/dL; HDL-c, 51 mg/dL; LDL-c, 87 mg/dL; TG, 75 mg/dL; HbA1c, 5.6%; and CRP, 0.55 mg/dL. The patient has hypertension and takes medicine to control blood pressure. However, the patient did not present metabolic syndrome and type 2 diabetes. Another p.Ser36Thr variant carrier was previously described by our group (Fonseca et al., 2019), accounting two p.Ser36Thr variant carriers identified in the total Brazilian cohort (MAF: 0.003).

We also have identified one patient with the p.Ala175Thr variant (MAF: 0.003). This pathogenic variant was described in a 52 years old female patient with morbid obesity, metabolic syndrome, and hypertension. Her current weight was 130.6 kg; height, 1.56; BMI, 53.7 kg/m^2^; waist circumference, 131 cm; hip circumference, 158 cm; neck circumference, 41 cm; BAI, 63.1% and WHR, 0.83. Regarding her biochemical measurements, FBG was 94 mg/dL; TC, 192 mg/dL; HDL-c, 39 mg/dL; LDL-c, 137 mg/dL and TG, 78 mg/dL. The patient did not exhibit type 2 diabetes.

The available data of both cases were summarized in Supplementary Table 1.

## Discussion

MC4R integrates the melanocortinergic pathway related to energy homeostasis. During food intake, increased amounts of α-MSH and cocaine and amphetamine-regulated transcript (CART) are secreted and bind to MC4R in the paraventricular nucleus of the hypothalamus, promoting the satiety and increasing energy expenditure. On the other hand, during the low energy state, an increase in appetite and a decrease in energy expenditure is promoted by the neuropeptide AgRP (agouti-related peptide), a MC4R antagonist (da Fonseca et al., 2017). Since 1998, when the first variants at the *MC4R* gene were detected in obese cases (Vaisse et al., 1998; Yeo et al., 1998), more than 350 variants have been described. Among them, 69 variants were predicted to be pathogenic or likely pathogenic, leading to a melanocortinergic pathway disruption (Collet et al., 2017b; Fairbrother et al., 2018). MC4R impairment is the most common form of monogenic obesity (6% of obesity cases) and was reported to have a codominant mode of inheritance. Furthermore, modifications in the *MC4R* gene were also associated with an increased in lean mass (Farooqi et al., 2003).

A previous *MC4R* screening performed by our group (Fonseca et al., 2019) had identified five variants (p.Met1?, p.Ser36Thr, p.Val103Ile, p.Ile98=, and p.Phe202Leu) in 16 subjects from a Brazilian cohort composed of 97 cases of childhood-onset obesity and 60 cases of adolescence/youth-onset obesity. Compared to non-carriers, p.Ser36Thr, p.Val103Ile, and p.Phe202Leu carriers had a higher WHR median and were associated with hypertension (*P* = 0.048 and *P* = 0.014, respectively). Cases with p.Val103Ile variant also showed lower diastolic blood pressure (DBP), lower systolic blood pressure (SBP), and hypertension (*P* = 0.020, *P* = 0.065, and *P* = 0.003, respectively). In the current study, we extend our cohort including 163 new patients in the screening of the *MC4R* gene. Altogether, 320 Brazilians with severe obesity were enrolled in this study cohort, in which 122 cases had childhood-onset, 79 cases reported adolescence/youth-onset and 119 patients declared adult-onset. Seven *MC4R* variants (p.Met1?, p.Ser36Thr, p.Val103Ile, p.Ala175Thr, p.Phe202Leu, p.Ile198=, and p.Ile251Leu) were detected in 25 individuals, accounting for a *MC4R* variant frequency of 0.045 (4.5%). We observed 14 variants in 12 patients with childhood-onset, 5 variants in 5 patients with adolescence-onset and 8 variants in 8 patients with adulthood-onset obesity.

**FIGURE 1:**
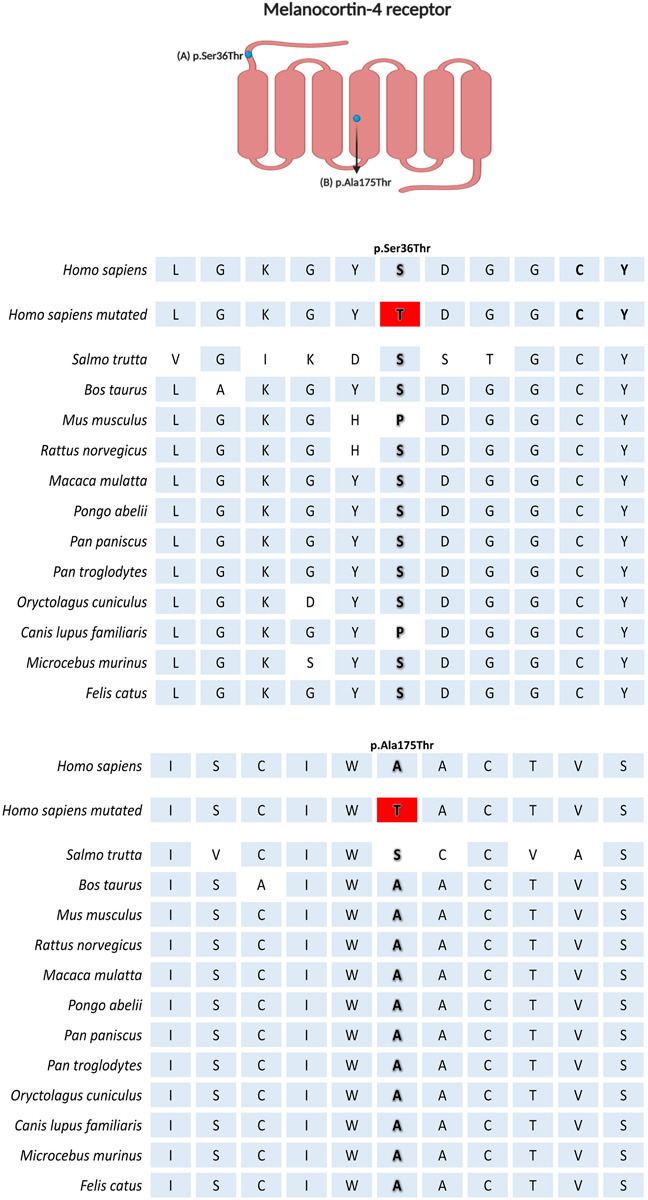
Multiple sequence alignment comparison of *MC4R* across the species by Clustal Omega (1.2.4). Created with BioRender.com.

Among the new cases, we have identified two potentially pathogenic variants (p.Ser36Thr and p.Ala175Thr) in our patients with adulthood-onset obesity. The p.Ser36Thr is an extremely rare mutation at the *MC4R* gene, which was reported in only four studies (Hughes et al., 2009; Bonnefond et al., 2016; Logan et al., 2016; Fonseca et al., 2019). Two female patients with similar BMI were found to carry this variant in our cohort (MAF: 0.003). One case, described previously (Fonseca et al., 2019), developed obesity during childhood and exhibited type 2 diabetes mellitus, metabolic syndrome, and hypertension. The new case we are reporting has adulthood-onset obesity and was diagnosed with hypertension. The frequency of this variant reported in the Genome Aggregation Database (gnomAD) is 3.97804^–06^ (exomes) and 3.18492^–05^ (genomes). This variant was absent in the 1000 Genomes and the ABraOM databases. In our Brazilian cohort, p.Ser36Thr was detected with a MAF of 0.003. Firstly, the p.Ser36Thr variant was detected by Hughes et al. (2009), who observed a frequency of 0.0005 in a Bantu cohort. The study screened the *MC4R* gene of 1.051 individuals from 51 populations [Human Genome Diversity Panel (HGDP-CEPH)], and also screened the *MC4R* gene in 11 primate species and 41 vertebrate species. The MC4R sequences of different species were compared to check whether the nucleotides substituted in the observed mutations are conserved in the molecular evolution of the gene. They observed a low *MC4R* variant diversity in humans, that was related to a negative selection as a result of the MC4R impairment caused by some non-synonymous mutations. Bonnefond et al. (2016) also observed the rare variant p.Ser36Thr in a Swiss cohort of 872 severely obese patients (MAF: 0.0006). In our analysis, the serine amino acid in the position 36 of MC4R proved to be conserved (Figure 1). Interestingly, the cAMP activity analysis showed a neutral role of p.Ser36Thr mutation (Larsen et al., 2005; Hughes et al., 2009; Logan et al., 2016). This variant was also identified in a single subject (BMI: 28.2 kg/m^2^) from a black South African cohort enrolling 187 individuals (MAF: 0.003) (Logan et al., 2016). Although it is suggested that *MC4R* p.Ser36Thr does not exhibit differential activity from its wild-type protein, this variant was mostly reported in overweight or obese individuals. Therefore, we suggest that the p.Ser36Thr should be investigated considering a possible impact on MC4R functionality.

To our knowledge, this is the first report of the p.Ala175Thr variant in the South American population. This variant was identified in one female case (MAF: 0.003) with morbid obesity and comorbidities. Yeo et al. (2003) detected this variant in one severely obese UK proband (MAF: 0.0015) with early-onset. Furthermore, two Italian cases were reported; one was detected in a male adult with severe early-onset obesity (Buono et al., 2005). Moreover, another case was one obese female patient (MAF: 0.005) with binging eating disorder (BED), who become obese after BED onset (Tortorella et al., 2005). One obese 175Thr allele carrier was also identified in a Danish cohort (MAF: 0.0007) (Larsen et al., 2005). To date, only one case-control study detected this variant in a lean subject from Caucasian origin (Calton et al., 2009). The alanine in position 175 of MC4R is a well-conserved amino acid during evolution (Figure 1). Functional studies of the p.Ala175Thr variant receptor are controversial. Yeo et al. (2003), observed close-to-average binding affinity to NDP-MSH and AgRP agonists but with reduced cAMP activity. Farooqi et al. (2003), also related a partial MC4R activity conferred by the mutant allele. Additionally, Alfieri et al. (2010), also identified that the receptor had a decreased activity in 30% due to a dominant-negative effect exerted by the minor allele. Controversially, Xiang et al. (2006), observed a similar pharmacological profile to its wild-type, with similar NDP-MSH agonist stimulation and modest increased binding affinity. Furthermore, Stäubert et al. (2007) showed that the p.Ala175Thr variant occurs naturally in fish *Mc4r* ortholog and cAMP had similar values from wild-type. Therefore, more studies are needed to elucidate the impact of p.Ala175Thr on MC4R function. This information will allow the researchers/clinicians to interpret obesity in a more individual manner, allowing for specific medical management.

Bariatric surgery in association with pharmacotherapy remains the gold standard treatment for severe obesity. However, several anti-obesity drugs may cause deleterious side effects (reviewed on Coulter et al., 2018). Selective MC4R agonists have been investigated for their effectiveness in weight regulation (Kühnen et al., 2019). The first-class synthetic MC4R agonist (LY2112688) displays adverse effects such as increased SBP (Maier and Hoyer, 2010). The second-generation MC4R agonist, setmelonotide (RM-493), described by Kievit et al. (2013), was successful in reducing food intake and decreasing body weight, in addition to improving glucose homeostasis, insulin sensitivity, and leptin levels, without causing blood pressure-related adverse effects to diet-induced obese Rhesus macaques (Kievit et al., 2013). The first study to administer setmelanotide to obese humans (Chen et al., 2015) observed that, after 72 h of agonist administration, the treated subjects showed increased resting energy expenditure (6.4%) and fat oxidation. Also, the authors did not observe any blood pressure complications. A human clinical trial, by Collet et al. (2017a), tested setmelanotide efficacy in *MC4R* variant obese carries. The promising MC4R agonist was able to induce a weight loss of approximately 0.6 kg/week over 4 weeks, besides no increase in heart rate or blood pressure. Setmelanotide treatment was also capable to promote weight loss in subjects with pro-opiomelanocortin (POMC) and leptin receptor (LEPR) deficiency, making this synthetic peptide a promising drug to treat obese subjects identified with variants in leptin-melanocortin pathway (Clément et al., 2018). In this context, functional studies are necessary to elucidate how identified variants in this study lead to or increase the susceptibility to obesity. These results would be prerequisite for treating potential patients with MC4R targeting drugs.

This study had some limitations that may be considered when analyzing the our results: (1) it was not possible to perform the segregation analysis, since family members were not available; (2) the period of obesity onset was self-reported; (3) functional analyses were not carry out in order to investigate the impact of the found genetic variants on protein structure and function.

## Conclusion

In the present study, we have identified five *MC4R* variants in patients with severe obesity, with a total prevalence of 3.7%. Among these genetic variants, we have found one potentially pathogenic variant (p.Ala175Thr) in a patient with extreme obesity, who developed this phenotype during adulthood. The p.Ala175Thr, a variant with reported impacts on MC4R activity, is documented here for the first time in a Brazilian cohort. Our study supports the importance of genetic testing to detect patients who could benefit from molecular diagnostics, genetic counseling, and specific medical management, such as setmelanotide, that has been tested as a specific anti-obesity drug to treat obese subjects with *MC4R* polymorphism, evading adverse effects, which are related to other pharmacological treatments.

## Data Availability Statement

The raw data supporting the conclusions of this article will be made available by the authors, without undue reservation, to any qualified researcher.

## Ethics Statement

This study protocol was approved by the Oswaldo Cruz Foundation Ethics Committee (CAAE: 09225113.0.0000/Protocol n°: 346.634). Written Informed consent was obtained from all participants (resolution n° 466/2012 of Ministry of Health, Brazil). The patients/participants provided their written informed consent to participate in this study.

## Author Contributions

AF, PC, GC, and PB: conception and design of the study. KS, GS, and AF: drafting the article and acquisition of data. JN, FM, and JC: acquisition of data. KS, AF, GA, and LP: analysis and interpretation of data. KS: wrote the manuscript. VZ, MC, FK, ER, and CM-M: revising it critically for important intellectual content. All authors read and approved the final version.

## Conflict of Interest

The authors declare that the research was conducted in the absence of any commercial or financial relationships that could be construed as a potential conflict of interest.

## Footnotes

1 https://folding.biofold.org/i-mutant/i-mutant2.0.html

2 http://mupro.proteomics.ics.uci.edu/

3 https://www.ncbi.nlm.nih.gov/

4 https://gnomad.broadinstitute.org/

5 http://www.internationalgenome.org/

6 http://abraom.ib.usp.br/

7 https://www.ebi.ac.uk/Tools/msa/clustalo/
