# Supplementary material for: Identification of a Rare and Potential Pathogenic MC4R Variant in a Brazilian Patient With Adulthood-Onset Severe Obesity
Source: Front Genet. 2020 Dec 9;11:608840. doi: 10.3389/fgene.2020.608840 (PMC7756028; doi:10.3389/fgene.2020.608840)
Supplement: Supplementary file 1 [file Table_1.DOCX]

Supplementary Material

| **Supplementary Table 1.** Anthropometric and serum biochemistry profile and blood pressure characterization of the new cases | | |
| --- | --- | --- |
| **Variables** | **Carriers** | |
|  | **p.Ser36Thr** | **p.Ala175Thr** |
| **Age (years)** | 42 | 52 |
| **Gender** | Female | Female |
| **Weight (kg)** | 123.6 | 130.6 |
| **Height (m)** | 1.63 | 1.56 |
| **BMI (kg/m^2^)** | 46.5 | 53.7 |
| **BAI** **(%)** | 53.6 | 63.1 |
| **Waist circumference (cm)** | 142.5 | 131 |
| **Hip circumference (cm)** | 149 | 158 |
| **Neck circumference** | 40 | 41 |
| **WHR** | 0.96 | 0.83 |
| **Blood pressure (mm Hg)** | 99/62 | NA |
| **FPG (mg/dL)** | 69 | 94 |
| **Cholesterol total (mg/dL)** | 153 | 192 |
| **HDL-cholesterol (mg/dL)** | 51 | 39 |
| **LDL-cholesterol (mg/dL)** | 87 | 137 |
| **Triglycerides (mg/dL)** | 75 | 78 |
| **HbA1c (%)** | 5.6 | NA |
| **CRP (mg/dL)** | 0.55 | NA |
| **Hypertension** | Yes | Yes |
| **Metabolic syndrome** | No | Yes |
| **Type 2 diabetes** | No | No |
| Abbreviations: BAI, body adiposity index; BMI, body mass index; CRP, C-reactive protein; FPG, Fasting Plasma Glucose; HDL-cholesterol, high-density lipoprotein-cholesterol; LDL-cholesterol, low-density lipoprotein-cholesterol; HbA1c, glycated hemoglobin; WHR, waist–hip ratio; NA, data not available. | | |
